# Technical report: 3D printing of the brain for use as a visual-aid tool to communicate MR imaging features of hypoxic ischaemic injury at term with non-physicians

**DOI:** 10.1007/s00381-018-3838-2

**Published:** 2018-05-26

**Authors:** Savvas Andronikou, Ewan Simpson, Maciej Klemm, Schadie Vedajallam, Anith Chacko, Ngoc Jade Thai

**Affiliations:** 1 Department of Pediatric Radiology, Bristol Royal Hospital for Children, University of Bristol, Upper Maudlin Street, Bristol, BS2 8BJ UK; 2 Department of Electrical and Electronic Engineering, University of Bristol, Bristol, UK; 3 CRICBristol, University of Bristol, Bristol, UK

**Keywords:** Technical report, MR imaging features, Hypoxic ischeamic injury

## Abstract

3D printing has been used in several medical applications. There are no reports however of 3D printing of the brain in children for demonstrating pathology to non-medical professionals such as lawyers. We printed 3D models of the paediatric brain from volumetric MRI in cases of severe and moderate hypoxic ischaemic injury as well as a normal age matched control, as follows: MRI DICOM data was converted to NifTI (Neuroimaging Informatics Technology Initiative) format; segmentation of the brain into CSF, grey, and white matter was performed; the segmented data was converted to STL format and printed on a commercially available scanner. The characteristic volume loss and surface features of hypoxic ischaemic injury are visible in these models, which could be of value in the communication of the nature and severity of such an insult in a court setting as they can be handled and viewed from up close.

## Background

Anatomic medical 3D printing is useful for education of students, informed consenting and forensic evidence in court [1, 2]. 3D prints from delayed MR scanning of term hypoxic ischaemic could be used to display the characteristic watershed cortical volume loss [3]. This would allow lawyers, judges and jury members to hold, turn and feel the surface of the brain rather than expecting them to understand hundreds of MRI slices on screen. MRI, CT or 3D ultrasound data can all produce a 3D model. Advantages of graspable 3D prints is that tactile feedback gives a better understanding of anatomy than 2D or 3D images on a screen [1]. We describe a 3D printing technique from MRI generating physical models of the brain for clinical and medico-legal purposes.

## Description

MRI scans of 2 age-matched children with previous peri-natal term partial-prolonged hypoxic ischaemic injury were selected from a research database: one with severe injury and one with selective anterior watershed injury. The MRI scan of a normal age-matched child was also selected.

3D T1 DICOM data was converted to NifTI format in open source software ‘dcm2nii’ (nitrc.org/projects/dcm2nii - accessed 01/10/2016). Automated skull stripping and segmentation of the brain were performed using Statistical Parametric Mapping v.12 (SPM12) and VBM8 toolbox v.445 in MATLAB (The MathWorks, Inc., 2014, Natick, Massachusetts, USA). This is a voxel-based morphometry approach that is objective and automated [4]. The tissue segmentation step in VBM8 uses an adaptive Maximum A Posterior (MAP) technique without the need for a priori information about tissue. The segmentation process produces maps of grey matter, white matter and CSF; the fidelity of the grey matter/CSF boundary segmentation is of greatest importance in creating an accurate 3D model of the brain surface, whereas grey/white matter segmentation is less important for creating surface models because these files are combined prior to exporting the segmented file to the mesh creation stage. Segmented brains should be visually compared to the standard T1 images, and parameters (variable for different MRI scanners) can be adjusted—ideal values can be achieved by defining the bias field correction in VBM8 software. Values used for the three selected brains are summarised in Table 1.

**Table 1:** ᅟValues used for the three selected brains

	NMD001 (normal)	MP-000175-01 (mild abnormality)	CASE002 (severe abnormality)
Segmentation: package	SPM12	VBM8	VBM8
Segmentation: bias regularisation	0.0001 (very light)	0.00001 (extremely light)	0.00001 (extremely light)
Segmentation: bias FWHM cutoff	60 mm	40 mm	40 mm
Conversion to STL: sampling points	Original segmentation image	Resampled (× 2 sample points)	Resampled (× 2 sample points)
Conversion to STL: node points	400,000	100,000	100,000
Conversion to STL: target maximum tetrahedral element volume	0.5	1	0.2
Conversion to STL: poisson surface reconstruction	Yes	Yes	Yes
Printing: % infill	20%	10%	20%
Printing: number of shells	3	3	3

Segmented NifTI files are then converted to a virtual ‘mesh’ of tetrahedral elements to give a representation of the brain surface, which is exported as a stereolithographic (.stl) data file for 3D printing. We used iso2mesh (http://iso2mesh.sourceforge.net/cgi-bin/index.cgi - accessed 13/01/2017), a free Matlab based toolbox, to combine the grey and white matter files and generate the STL file. At this stage, the number of sampling points and the number of node points generated in the mesh can be modified after which surface mesh smoothing is applied. In general, there is a trade-off between mesh quality (number of nodes, maximum tetrahedral element volume, etc.) and processing time. Now the 3D model can be printed from the STL file. We used a commercially available MakerBot Replicator 2 Desktop 3D printer (Makerbot Industries LLC, Brooklyn, NY), using ColorFabb polylactic acid (PLA). Layer height (contributing to resolution) was set to 0.2 mm; infill (the internal scaffold which contributes to structural integrity) was set to 20%; and shell number (also a determinant of structural integrity) set to 3 (Table 1).

Printed models were examined for demonstration of pathology by comparing to cross-sectional images and differences between each other.

Ethical approval falls under two larger projects.

## Results

**Fig. 1:**
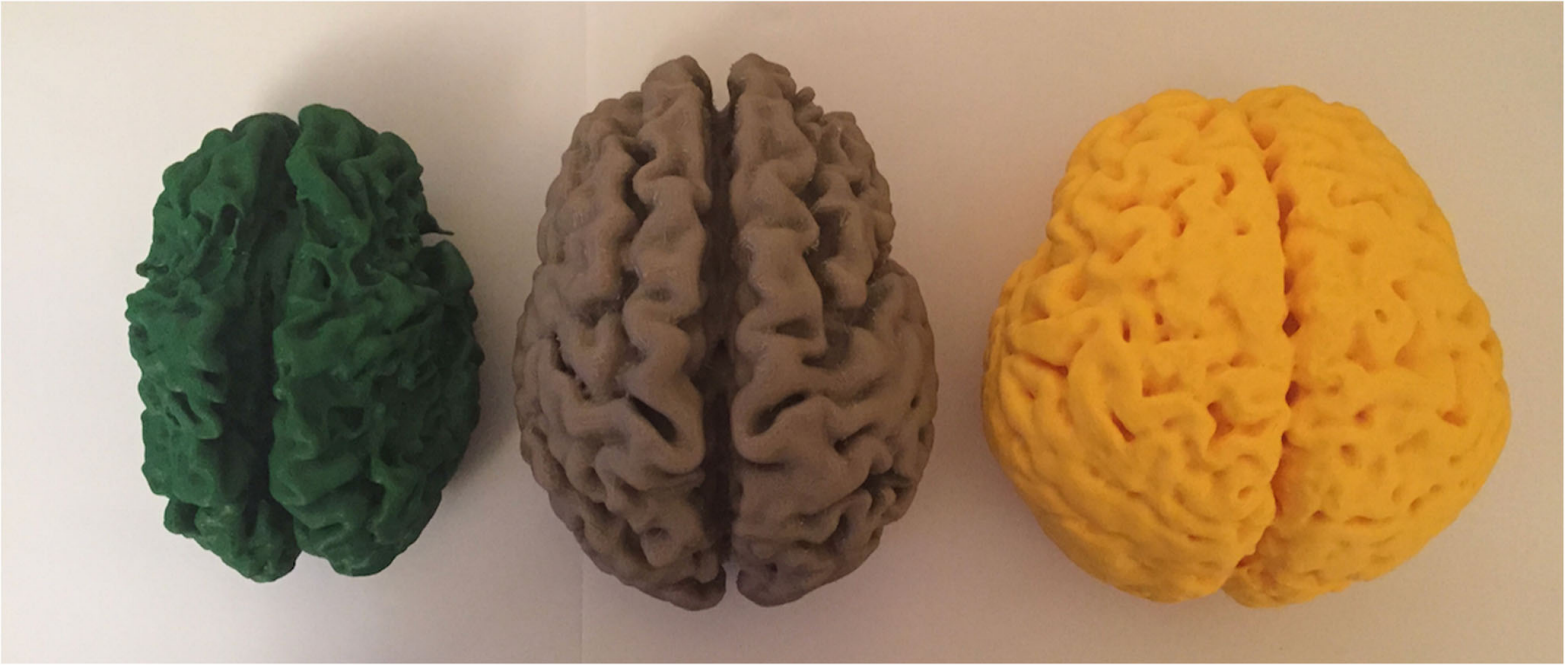
3D printed models of the brains of age matched children. On the left, a child severely affected by partial-prolonged hypoxic ischaemic injury [GREEN brain]; in the middle, a child with mild/moderate hypoxic ischaemic injury predominantly affecting the parasagittal and anterior watershed zones [GREY brain]; on the left a healthy brain [YELLOW brain]. When printed at scale and placed side-by-side, the extent of volume loss due to atrophy is far more apparent than when viewing axial images on an MRI because they allow appreciation of scale

**Fig. 2:**
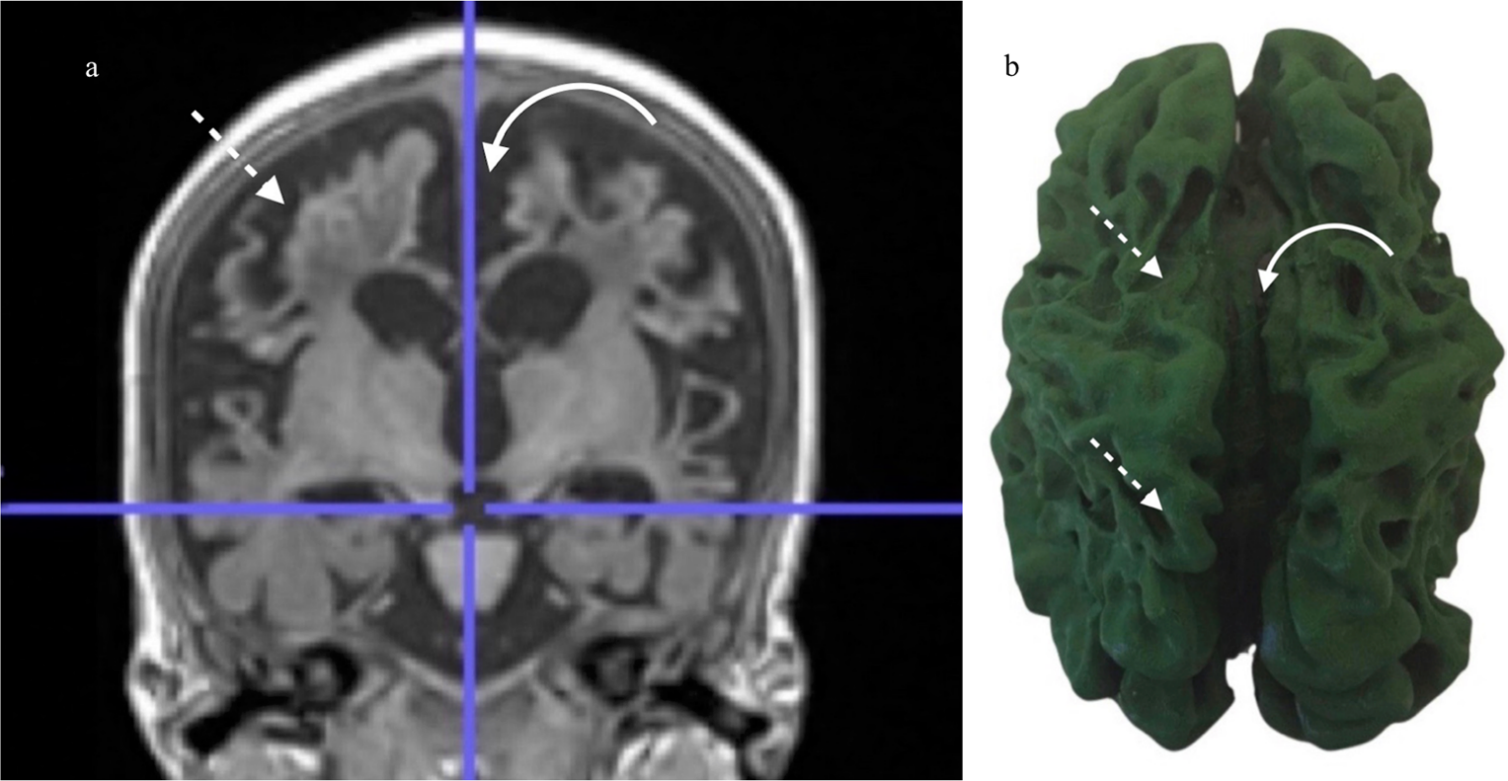
**a**, **b** Severe partial-prolonged hypoxic ischaemic injury to the brain with MR imaging at the age of 4 years and accompanying 3D print. **a** Coronal T1 weighted MRI at the level of the 3rd ventricle in a child with changes of severe term partial-prolonged hypoxic ischaemic injury, demonstrating marked widening of the interhemispheric fissure (curved arrow) with severe bilateral, symmetric volume loss in the anterior watershed distribution as well as perisylvian regions (dashed arrow). **b** 3D printed model of the brain shown in Fig. 3a demonstrating the pronounced interhemispheric and sulcal widening due to the volume loss (curved arrow) and severe bilateral, symmetric shrinkage of the brain along the peri-Sylvian and peri-Rolandic regions (dashed arrows)

**Fig. 3:**
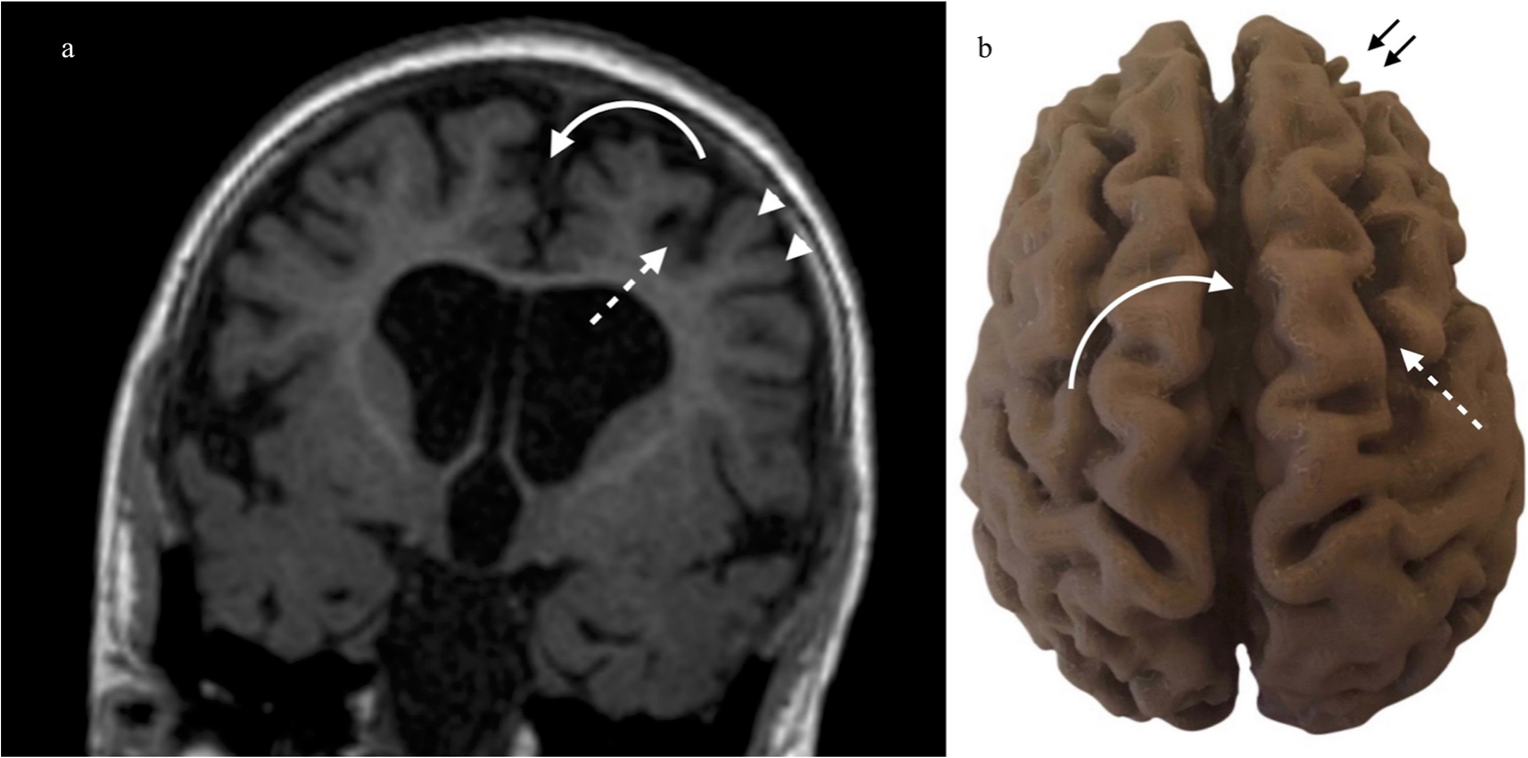
**a**, **b** Moderate/mild partial-prolonged hypoxic ischaemic injury to the brain with MR imaging at the age of 4 years and accompanying 3D print. **a** Coronal T1 weighted MRI at the level of the third ventricle demonstrates relatively mild changes of term partial-prolonged hypoxic ischaemic injury. Note widening of the interhemispheric fissure (curved arrow) and of the parasagittal sulci (dashed arrow) with ulegyria (arrowhead). **b** 3D printed model of the brain shown in Fig. 4a (mild changes of term partial-prolonged hypoxic ischaemic injury). There is moderate widening of the interhemispheric fissure (curved arrow). Note also prominent widening of the superior frontal sulcus corresponding to a parasagittal and anterior watershed continuum of volume loss (dashed arrow). Ulegyria is seen anteriorly (small black arrows)

**Fig. 4:**
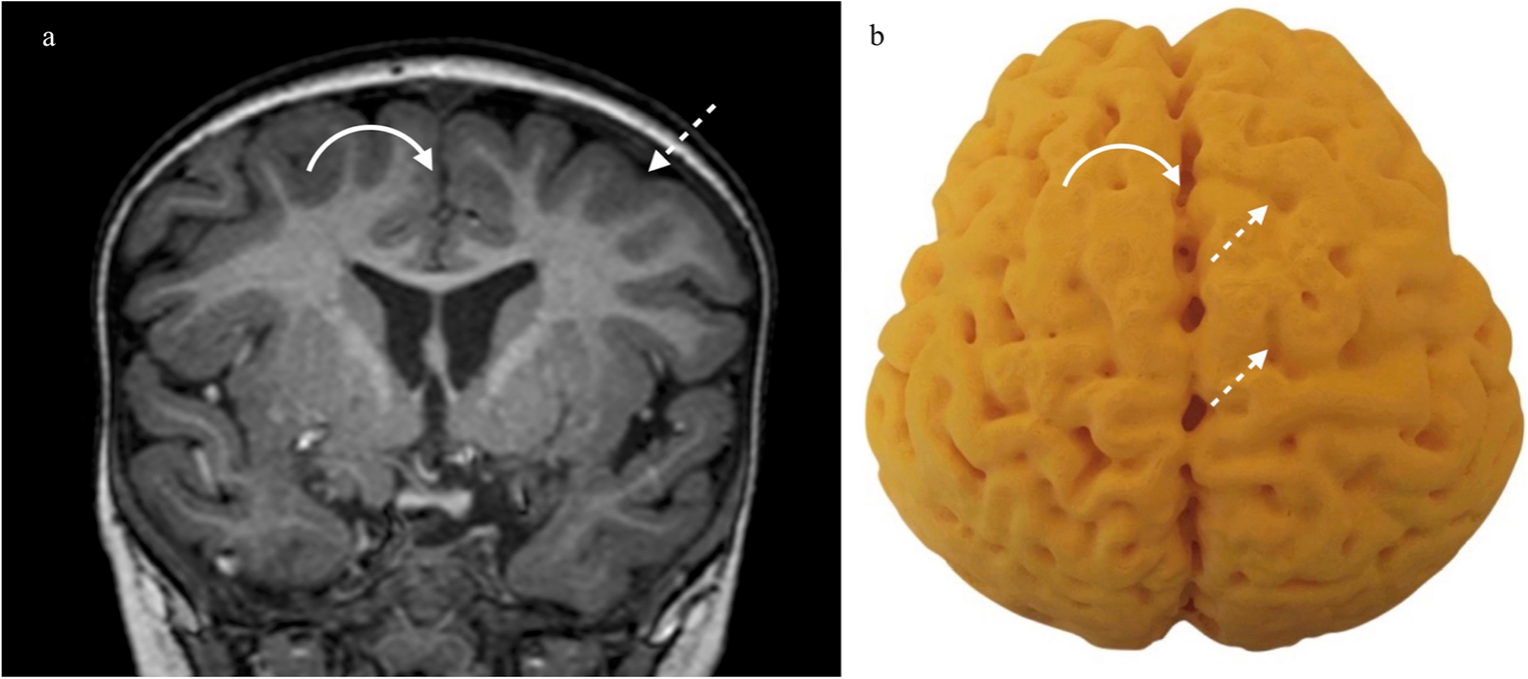
**a**, **b** Control patient with ‘normal’ MR imaging at the age of 4 years and accompanying 3D print. **a** Coronal T1 weighted MRI at the level of the 3rd ventricle in a healthy child age-matched with those in Figs. 3 and 4. The interhemispheric fissure is narrow (curved arrow) and the hemispheres and gyri are closely apposed (dashed arrow). **b** 3D printed model of the brain. The interhemispheric fissure is narrow (curved arrow) and the hemispheres and gyri are closely apposed (dashed arrows)

Three representative 3D models were achieved (Figs. 1, 2, 3, and 4). Duration of printing ranged between 19 and 22 h. When viewed together (Fig. 1), the most striking observation was the decrease in overall volume of brains with hypoxic injury when compared to the normal printed at scale—not easily appreciated on cross-sectional images.

Pathologic brains also demonstrated characteristic regional prasagittal atrophy in with widening of interhemispheric fissures and sulci as well as ulegyria, i.e. gyri thinner at the depth of the sulci than the surface (Figs. 2 and 3). This correlated well with the cross-sectional imaging (Figs. 2 and 3). In contrast, the interhemispheric fissure and sulci were closely apposed in the normal brain without ulegyria (Fig. 4).

## Discussion

3D printing is increasingly used in medicine for education, communication in pre-operative patient consenting, customised surgical planning, simulation for teaching new surgical techniques, production of surgical implants and bio-printing of scaffolds for new organs [1]. In addition, 3D prints of anatomic structures from imaging data have been used as forensic evidence, i.e. given to jurors who can hold and rotate them to view from any angle [2].

In the context of term partial-prolonged hypoxic ischaemic injury, imaging performed months or years after an insult demonstrates characteristic regional cortical volume loss in the watershed regions which result in prominent surface markings [3]. Delayed imaging demonstrating atrophy is performed when parents seek compensation [5]. However, lawyers, judges and jury members who rely on ‘conclusive’ diagnostic imaging features for arguing each case have no training in the interpretation of multi-planar, multi-sequence, cross-sectional MRI images—which even neuroradiology subspecialists show variable reliability in interpreting [6]. With 3D printed brains, a non-radiologist can understand the regional surface cortical shrinkage associated with hypoxic ischaemic injury in term neonates and appreciate brain volume differences at scale when comparing against age matched controls. Our 3D prints compared well with source images in accurately demonstrating normal and abnormal surface markings in children with mild and severe partial-prolonged hypoxic ischaemic injury.

## Conclusion

3D printing of brain MRI scans successfully demonstrated regional cortical volume loss and altered cortical morphology associated with partial-prolonged hypoxic ischaemic injury at term, additionally allowing appreciation of overall brain size against normal. 3D prints could prove useful tool for communicating such findings in court, as well as in discussion with parents and trainees.
